# Clinicoepidemiological Observational Study of Acquired Alopecias in Females Correlating with Anemia and Thyroid Function

**DOI:** 10.1155/2016/6279108

**Published:** 2016-01-20

**Authors:** Kirti Deo, Yugal K. Sharma, Meenakshi Wadhokar, Neha Tyagi

**Affiliations:** Department of Dermatology, Dr. D. Y. Patil Medical College and Hospital and Research Centre, Pimpri, Pune, Maharashtra 411018, India

## Abstract

Alopecia can either be inherited or acquired; the latter, more common, can be diffuse, patterned, and focal, each having cicatricial and noncicatricial forms. This observational study of 135 cases in a semiurban Indian population aimed to detect the prevalence of various forms of acquired alopecia in females and correlate the same with levels of hemoglobin, serum ferritin, triiodothyronine, thyroxin, and thyroid stimulating hormone. The majority (84, 62.2%) of our cases of alopecia had telogen effluvium followed by female pattern alopecia (32, 23.7%). Stress (86, 63.7%), topical application of chemicals (72, 53.3%), systemic medications for concurrent illnesses (62, 5%), and pregnancy (14, 10.3%) were the common exacerbating factors. Neither low hemoglobin (<12 gm%, 73.4%) nor low serum ferritin (<12 *μ*g/L, 6.7%) was found to be statistically significant. A majority (90, 90.9%) of 99 cases with anemia (hemoglobin levels of <12 gm%) had serum ferritin levels >12 *μ*g/L. Though lack of vitamin B12 testing was a limitation of our study, its deficiency could be the probable cause of iron deficiency as the majority (58, 64.4%) of these cases, as indeed majority (89, 65.4%) of our study population, were vegetarians. Thyroid disorders (23, 17%, including 9 newly diagnosed) were not of significance statistically.

## 1. Introduction

The importance of human hair in social communication and sexual attraction is enormous, its loss (alopecia), shaft defects, or excess on the body are often accompanied by diminished sense of personal well-being, self-esteem, depressive moods, and withdrawal from society [1]. Alopecia, due to its multiple, virtually impossible to pinpoint, underlying causes and poor response to treatment, remains one of the most common entities that baffle dermatologists. Patient's anxiety compounds the scenario by causing increased hair loss on one hand and lack of response to treatment on the other. Lack of societal acceptance of hair loss in women, unlike in men, generates distress [2] leading not only to low self-esteem, poor body image, feeling of guilt, difficulty sleeping, and restriction in active social life [3] but also to overpresentation of women amongst those seeking treatment [4].

Alopecia can either be inherited or acquired; the latter, more common, is broadly classified into diffuse, patterned, and focal types, each further divided into scarring (cicatricial) and nonscarring (noncicatricial) forms [1]. The relatively rare inherited forms result either from improper development or nondevelopment of follicles or due to a defective hair shaft; the former has been divided again into focal (e.g., aplasia cutis congenita) or diffuse (e.g., ectodermal dysplasia) forms. The acquired hair shaft defects and increased hair breakage, commonly as a result of hair grooming practices, are reversible on cessation thereof and the inherited ones, though irreversible, also tend to improve with the advancing age [1]. Though the diagnosis of hair loss is mainly clinical, additional techniques like the hair pull test, video-dermoscopy, and histopathology are used to supplement history and examination.

The controversy regarding the association between nutritional factors and hair loss continues to persist. The supportive management with zinc and iron supplements is empirical rather than being based on unambiguous data regarding role of low serum zinc concentrations or deficient serum ferritin. As excessive intake of nutritional supplements may actually lead to increased hair loss, it appears logical to prescribe them in cases only with the proven deficiency [5].

This study aimed to detect the prevalence of various forms of acquired alopecia in females and their correlation with anemia and triiodothyronine (T3), thyroxin (T4), and thyroid stimulating hormone (TSH) levels.

## 2. Materials and Methods

This observational study comprised 135 consecutive female patients presenting to our department for the treatment of alopecia during the period of October 2014 to July 2015. Institutional ethical committee clearance and an informed consent of each patient were obtained and detailed history and examination undertaken to assess the type of alopecia, exacerbating factors, and associated systemic illnesses. Patients with diffuse hair loss, presence of one or more of the exacerbating factors, namely, stress, pregnancy, and application of chemicals, and positive hair pull test were considered to have telogen effluvium. Patients with reduction of number of hair, especially in biparietal, bitemporal, and vertex regions with preservation of anterior hair implantation line, were considered to have female pattern hair loss. However, overlapping of both of these diseases could occur in some cases. All patients were tested for hemoglobin (Hb), serum ferritin, and T3, T4, and TSH levels. The data obtained were analyzed statistically using SPSS version 22.0. Correlation between variables was calculated using Spearman's rank correlation coefficient and Chi-square test. A value of *p* < 0.05 was considered significant.

## 3. Results

The age of our study patients ranged from 15 to 60 (mean: 30.96 ± 8.489) years, with the majority (68, 50.4%) belonging to the third decade (Table 1). After onset of alopecia patients took one to 84 (mean: 15.64 ± 16.003) months to seek consultation, the largest segment (29, 21.5%) reporting after 12 months resulting in a significant, but mild positive, correlation (rho = 0.219; *p* = 0.011); OPD consultation gets increasingly delayed with the advancing age of the patients.

The first and second common types of alopecia in our study were telogen effluvium (TE) (84, 62.2%) and female pattern hair loss (FPHL) (32, 23.7%) (Table 1). The exacerbating factors found across the spectrum of alopecia were stress, in 86 (63.7%) patients; application of chemicals such as mehndi or dye, in 72 (53.3%); pregnancy and/or delivery six months preceding the presentation, in 14 (10.3%). Sixty-two (45.9%) patients were undergoing treatment for associated systemic illness(es): 21 (15.6%) for diabetes mellitus; 14 (10.4%) for polycystic ovarian disease; 11 (8.1%) for hypertension; 8 (5.9%) for hypothyroidism; 6 (4.4%) for hyperthyroidism; and 1 (0.7%) for tuberculosis and cancer each. Dandruff seen in 63 (46.7%) and oily scalp in 75 (55.6%) were not statistically significantly associated. The majority (86, 63.7%) of patients had diffuse hair loss followed by hair loss over the vertex (28, 20.7%) (Table 2). Family history of alopecia was given by only 6 (4.4%). FPHL, found in 8 (25%) postmenopausal cases in our study, revealed a statistically significant association (*p* = 0.006, strength of association, *χ* ^2^ = 21.36). Characteristic nail pitting seen in 9 (52.9%) of 17 cases of alopecia areata was also associated significantly (*p* = 0.001, strength of association, *χ* ^2^ = 0.545).

Hemoglobin levels ranged from 7.20 to 13.40 (mean: 10.97 ± 1.32) gm%; anemia (Hb < 12 gm%) was present in 99 (73.4%). Serum ferritin levels ranged from 5.10 to 200.70 (mean: 54.73 ± 41.5) *μ*g/L. Iron deficiency was seen in 9 (6.7%) of our study population having serum ferritin < 12 *μ*g/L; neither hemoglobin nor serum ferritin levels were statistically significant. Thyroid disorders present in 23 (17%) patients, including nine (6.7%) newly diagnosed ones, too, were not of statistical significance (Table 3).

## 4. Discussion

Despite its exact prevalence being difficult to estimate due to its subclinical nature [1], telogen effluvium is generally considered to be the most common type of alopecia in females followed by FPHL and alopecia areata [6]. Similar was the frequency of occurrence of these three types of alopecia in our study.

Telogen effluvium in women commonly occurs during the age of 30–60 years, usually commencing abruptly with or without a recognizable initiating factor [7]. Among the 84 patients of TE in our study, as also in a South Arabian one, its peak incidence (47, 56%) was during the third decade of life [7]. For unknown reasons, chronic TE seems to affect only women [7]. History of hair coming out along with the bulbs given by 74 (88.1%) of our 84 patients of TE was statistically significant (*p* = 0.00, strength of association, *χ* ^2^ = 24.180); 10 (11.09%) of these developed it after pregnancy; 58 (69.1%) underwent a period of stress during the preceding six months and 53 (65.1%) were vegetarians. Positive hair pull test in all scalp areas indicative of associated TE was detected in 46 (54.8%) of these 84 cases; the Saudi Arabian study reported positive hair pull test in 61% of their cases of TE [7]. Sixty-three (75%) of our 84 patients of TE were anemic (Hb < 12 gm%) and 8 (9.5%) were iron deficient (serum ferritin < 12 *μ*g/L).

Female pattern hair loss (FPHL) was observed in 32 (23.7%) patients in our study, 11 (34.4%) belonging to the third decade; unlike in most of the earlier studies, its prevalence was not seen to increase with age (Table 4) [8–11]. However, a comparison between prevalence studies is hampered by the lack of universally accepted criteria for diagnostic definition of this disease. Despite onset of FPHL during the reproductive years, there is a greater demand for its treatment among patients aged 25–40 years possibly explaining the preponderance of cases (21 of 32, 65%) aged 21–40 years in our study [12]. Lesser prevalence with increasing age in our study could be due to only 2 (1.48%) cases belonging to the last decade of the age of our study population. Hair loss over the vertex was present in 22 (68.8%) patients followed by that on the temporal zone (7; 21.9%) and diffuse hair loss (3; 9.4%). In an Australian study, the majority (64.4%) of such patients exhibited bitemporal hair loss [11]. Twenty (62.5%) of our cases of FPHL gave history of application of mehndi/hair dye; 20 (62.5%) of irregular menses or menopause; and 15 (46.1%) of stress. Hypertension and diabetes mellitus were present in 8 (25%) and 7 (21.9%) cases, respectively. Hair pull test was positive in 15 (46.1%); 20 (62.5%) were anemic and 1 (3.1%) was iron deficient, reaffirming the lack of role of nutrition in causation of FPHL. Insignificant (1, 3.1%) incidence of positive family history in our FPHL patients, unlike that of 45.2% (Wang et al.) [8] and 19.2% (Paik et al.) [9], could probably be due to different, as yet unknown, underlying genetic factors in different ethnicities.

The prevalence (17, 12.6%) of alopecia areata (AA) in our study was almost half of that (25%) found in a the United States based study by McMichael et al. Unlike majority (11; 64.1%) of these patients in our study belonging to the third decade, majority of them in study by McMichael et al. belonged to 4th to 6th decades of life [13]. In a German study its highest prevalence was seen during 2nd to 4th decades of life, up to 66% of these patients being below 30 years of age and only 20% older than 40 years [14]. The onset below the age of 40 was reported in 70% of the patients of AA in a Greek study by Kyriakis et al., 48% having onset of AA before the age of 20 years [15]. The appearance of first patch before the age of 20 was seen in 60% of cases in a United States based study by Wasserman et al. Surprisingly, just 2 (12%) of our cases of AA developed first patch before the age of 20 [16]. AA was associated with stress during the six months preceding its onset in 11 (64.7%) of our study patients, similar to that of Taiwanese (50%) and Romanian (45.6%) studies [17, 18], corroborating the well-known role of psychological stress in precipitating AA [19, 20]. However, family history of AA in our study was rarely positive (1, 5.9%), as also remarked in previous studies: 9% (Sharma et al.) [21] and 4.6% (Tan et al.) [22].

Anagen effluvium and frontal fibrosing alopecia each occurred only in one case in our study.

Hemoglobin levels ranged from 7.20 to 13.40 (mean: 10.97 ± 1.32) gm% in our study. Ninety-nine (73.4%) patients were found to be anemic (Hb < 12 gm%); 68 (50.3%) were mild (Hb 10–11.99 gm%), 31 (23%) moderate (Hb 7–9.99 gm%), and none severe (Hb < 7 gm%). This classification of anemia was based on Indian (national family health survey 3, 2005-2006) Government survey in which 48.3% (3890 of 8053) of women in Maharashtra, the Indian state of our study population, were found to be anemic [mild (32.8%); moderate (13.8%); severe (1.7%)] [23] suggesting anemia to be a major factor underlying hair loss. The prevalence of anemia in a study of 4032 asymptomatic females in the Indian state of Andhra Pradesh was found to be 52% [24]. In our study population, serum ferritin levels ranged from 5.10 to 200.70 (mean: 54.73 ± 41.46) *μ*g/L, nine (6.6%) being iron deficient (serum ferritin < 12 *μ*g/L), as compared to that of 33.3% in studies conducted in Bangladesh and Iran [24, 25]. Out of 99 patients of anemia, 9 (9.1%) were found to have iron deficiency anemia. Among the 90 non-iron deficiency anemia patients, 58 (63.1%) were vegetarians who probably had underlying vitamin B12 deficiency. In a study by Sinclair no direct relationship between low serum ferritin and hair loss was established and determination of serum ferritin as a routine investigation in women with chronic diffuse telogen hair loss was not found useful, doubting the role of iron supplementation in management of hair loss [26].

Twenty-three patients (17%) were found to be suffering from thyroid disorders: 13 (9.63%) were suffering from hypothyroidism and 10 (7.4%) hyperthyroidism, similar to the overall prevalence (18.6%) of thyroid disorders in Maharashtra [23] and much more than 2.5% of hypothyroidism and hyperthyroidism each in a German study [27]. However, 9 (6.7%) patients, 5 (3.7%) with hypothyroidism and 4 (3%) with hyperthyroidism, were newly diagnosed during our study (Table 3).

## 5. Conclusion

Telogen effluvium was the most frequent type of alopecia found in our study. Exacerbating factors included stress, pregnancy, and application of chemicals. Anemia detected in approximately 3rd/4th of our study population would appear as the commonest cause of alopecia. While only a small minority were diagnosed as having iron deficiency anemia, a majority suffered probably from vitamin B12 deficiency, being vegetarians. According to our study serum ferritin levels did not have significant role in the pathogenesis of hair loss and may not need to be estimated routinely. Women suffering from recalcitrant hair loss should undergo thyroid function tests to reveal subclinical thyroid disorders. Appropriate counseling and treatment can then be specifically directed at the etiology of hair loss, thus improving the patient outcome.

## Figures and Tables

**Table 1:** Distribution of alopecia in the age groups.

Age in years	Telogen effluvium	Alopecia areata	Female pattern hair loss	Frontal fibrosing alopecia	Anagen effluvium	Total
15–20	3	2	1	0	0	6
21–30	47	11	10	0	0	68
31–40	29	3	11	1	1	45
41–50	5	1	8	0	0	14
51–60	0	0	2	0	0	2
Total	**84**	**17**	**32**	**1**	**1**	**135**

**Table 2:** Location of alopecia.

Location	Telogen effluvium	Alopecia areata	Female pattern hair loss	Frontal fibrosing alopecia	Anagen effluvium	Total
Diffuse	83	0	3	0	0	86
Parietal	1	7	0	0	0	8
Temporal	0	4	7	0	1	12
Vertex	0	5	22	1	0	28
Occipital	0	1	0	0	0	1
Total	**84**	**17**	**32**	**1**	**1**	**135**

**Table 3:** Thyroid disorders throughout the spectrum of alopecia (new + old cases).

	Telogen effluvium		Alopecia areata		Female pattern hair loss		Frontal fibrosing alopecia	Anagen effluvium	Total
	N	O	N	O	N	O			
Hypothyroidism	2	4	1	1	2	3	0	0	**13**
Hyperthyroidism	3	5	1	1	0	0	0	0	**10**
Euthyroid	70		13		27		1	1	**112**
Total	**84**		**17**		**32**		**1**	**1**	**135**

N: new; O: old.

**Table 4:** Comparison between prevalence of female pattern hair loss.

Age in years	Wang et al. [8] (%)	Paik et al. [9] (%)	Birch et al. [10] (%)	Gan and Sinclair [11] (%)	Present study (%)
15–20	—	—	—	—	0.7
21–30	1.30	0.2	12.3	12.00	7.4
31–40	2.30	2.3	17.00	—	8.1
41–50	5.40	3.8	25.40	25.00	5.9
51–60	7.50	7.4	27.90	—	1.4
61–70	10.30	11.7	41.10	41.00	—
71–80	11.80	24.7	53.60	50.00	—
